# Chromosomal and DNA barcode analysis of the *Polyommatus* (*Agrodiaetus*) *damone* (Eversmann, 1841) species complex (Lepidoptera, Lycaenidae)

**DOI:** 10.3897/compcytogen.v15.i1.60347

**Published:** 2021-01-04

**Authors:** Vladimir A. Lukhtanov, Alexander V. Dantchenko

**Affiliations:** 1 Department of Karyosystematics, Zoological Institute of the Russian Academy of Sciences, Universitetskaya nab. 1, St. Petersburg 199034, Russia Zoological Institute of the Russian Academy of Sciences St. Petersburg Russia; 2 Faculty of Chemistry, Lomonosov Moscow State University, GSP-1, Leninskiye Gory 1/11, Moscow119991, Russia Lomonosov Moscow State University Moscow Russia

**Keywords:** *
Agrodiaetus*, chromosomal stasis, chromosome, *COI*, DNA barcoding, karyosystematics, taxonomy

## Abstract

The Polyommatus (Agrodiaetus) damone (Eversmann, 1841) species complex comprises from 5 to 8 species distributed in southeastern Europe and southern Siberia. Here we used chromosomal and DNA-barcode markers in order to test the taxonomic hypotheses previously suggested for this complex. We revealed that all taxa within this group demonstrate chromosomal stasis and share the same or very similar haploid chromosome number (n = 66 or n = 67). This finding is unexpected since the karyotypes are known to be very diverse and species-specific within the other taxa of the subgenus Agrodiaetus Hübner, 1822. Analysis of the mitochondrial gene *COI* revealed six diverged clusters of individuals within the complex. Each cluster has a specific geographic distribution and is characterized by distinct morphological features in the wing pattern. The clusters mostly (but not always) correlate with traditionally recognized species. As a result of our study, we describe a new subspecies P. (A.) iphigenides zarmitanus**subsp. nov**. from Uzbekistan and Tajikistan and show that the taxon originally described as Lycaena kindermanni var. melania Staudinger, 1886 represents a subspecies P. (A.) iphigenides melanius (Staudinger, 1886). Polyommatus (A.) samusi Korb, 2017 (**syn. nov**.) and P. (A.) melanius komarovi Korb, 2017 (**syn. nov**.) are considered here as junior subjective synonyms of P. (A.) iphigenides iphigenides (Staudinger, 1886).

## Introduction

The Polyommatus (Agrodiaetus) damone (Eversmann, 1841) species complex is a monophyletic group (Vershinina and Lukhtanov 2017) that comprises from 5 to 8 species distributed in SE Europe, Central Asia and S Siberia (Eckweiler and Bozano 2016). The taxa of the complex were previously revised by Staudinger (1899), Forster (1956, 1960), Dantchenko and Lukhtanov (1993) and Dantchenko (1997). There are also limited molecular (Wiemers 2003; Kandul et al. 2004, 2007; Lukhtanov et al. 2005, 2009; Vodolazhsky et al. 2011; Vodolazhsky and Stradomsky 2012) and chromosomal (Lukhtanov 1989; Kandul 1997; Lukhtanov et al. 1997; Lukhtanov and Dantchenko 2002a; Lukhtanov et al. 2005) data for a few taxa of the complex. However, the complex has never been systematically studied by using chromosomal and molecular markers, although such an approach is considered as an essential requirement for revealing taxonomic structure in the subgenus Agrodiaetus (Lukhtanov and Dantchenko 2002b; Kandul et al. 2004).

Here we analyzed karyotypes and mitochondrial DNA-barcodes of all species of the P. (A.) damone complex in order to test the taxonomic hypotheses previously suggested for this group (see the references above).

The taxa P. (A.) damone walteri Dantchenko et Lukhtanov, 1993, P. (A.) damone fabiani Bálint, 1997 and P. (A.) damone bogdoolensis Dantchenko et Lukhtanov, 1997 are not considered in this paper since neither chromosomal nor molecular data are available. This also applies to P. (A.) carmon altaiensis (Forster, 1956), recently treated by Eckweiler and Bozano (2016) as a separate species. All these taxa represent the most eastern populations of the P. (A.) damone complex distributed in Mongolia, Altai and southwestern Siberia. Morphologically they are close to other populations of *P. damone* or to *P. mediator* Dantchenko et Churkin, 2003. Their study will become possible in the future as soon as the material suitable for molecular and chromosomal analyses becomes available.

## Material and methods

### Molecular methods and DNA barcode analysis

Standard *COI* barcodes (658-bp 5' segment of mitochondrial *cytochrome oxidase subunit I*) were studied. *COI* sequences were obtained from 44 specimens representing the *P. damone* species group and from two samples [*P. damon* (Denis et Schiffermüller, 1775) and *P. icarus* (Rottemburg, 1975)] which were selected as outgroup (Table 1). Legs were sampled from these specimens, and sequence data from the DNA barcode region of *COI* were obtained at the Canadian Centre for DNA Barcoding (CCDB, Biodiversity Institute of Ontario, University of Guelph) using protocols described in Hajibabaei et al. (2005), Ivanova et al. (2006) and deWaard et al. (2008). Specimens examined are deposited in the Zoological Institute of the Russian Academy of Sciences, St. Petersburg, Russia and in the McGuire Center for Lepidoptera and Biodiversity (MGCL), Florida Museum of Natural History, University of Florida, Gainesville, Florida, USA. Photographs of these specimens, as well as collecting data are available in the Barcode of Life Data System (BOLD), projects Butterflies of Palearctic (BPAL) and Butterflies of Palearctic Part B (BPALB) at http://www.boldsystems.org/.

We also used 31 published *COI* sequences (Wiemers 2003; Kandul et al. 2004, 2007; Lukhtanov et al. 2005, 2009; Vodolazhsky et al. 2011; Vodolazhsky and Stradomsky 2012) which were downloaded from GenBank (Table 1).

**Table 1.** Specimens of the Polyommatus (Agrodiaetus) damone complex used in the DNA-barcode analysis.

Species and subspecies	Sequence code	Field code	GenBank number	Country	Locality	Reference
*P. damon*	FJ663230	n/a	FJ663230	Kazakhstan	Altai	Lukhtanov et al. 2009
*P. damone altaicus*	FJ663229	LOWA298-06	FJ663229	Kazakhstan	Saur-Tarbagatai Mts	Lukhtanov et al. 2009
*P. damone altaicus*	FJ663228	LOWA299-06	FJ663228	Kazakhstan	Saur-Tarbagatai Mts	Lukhtanov et al. 2009
*P. damone altaicus*	BPAL3395-16	CCDB-25452_F10	MW186990	Russia	Altai, Jarbalyk	This study
*P. damone altaicus*	BPAL838-11	CCDB-05724_G06	MW186700	Kazakhstan	Saur Mts, Saikan	This study
*P. damone altaicus*	BPAL839-11	CCDB-05724_G07	MW186701	Kazakhstan	Saur Mts, Saikan	This study
*P. damone altaicus*	AY496734	n/a	AY496734	Russia	Altai, Aktash	Kandul et al. 2004
*P. damone altaicus*	BPAL3394-16	CCDB-25452_F09	MW186989	Russia	Altai, Chemal	This study
*P. damone*	BPAL836-11	CCDB-05724_G04	MW186988	Russia	Volga, Volsk	This study
*P. damone*	BPAL837-11	CCDB-05724_G05	MW186992	Russia	Volga, Volsk	This study
*P. damone*	BPAL835-11	CCDB-05724_G03	MW186999	Russia	Volga, Akulovka	This study
*P. damone*	AY496735	n/a	AY496735	Russia	South Urals, Guberli Mts, Adaevo	Kandul et al. 2004
*P. damone irinae*	BPAL833-11	CCDB-05724_G01	MW186997	Russia	Volgograd Region, Olkhovka	This study
*P. damone irinae*	BPAL834-11	CCDB-05724_G02	MW186998	Russia	Volgograd Region, Olkhovka	This study
*P. damone irinae*	AY496736	n/a	AY496736	Russia	Volgograd Region, Olkhovka	Kandul et al. 2004
*P. damone pljushtchi*	AY496774	n/a	AY496774	Russia	Crimea, Ai Petri	Kandul et al. 2004
*P. damone* ssp.	BPAL524-11	n/a	MW186991	Kazakhstan	Karaganda Region, Akchatau	This study
*P. damone tanais*	BPAL825-11	CCDB-05724_F05	MW186993	Ukraine	Amvrosievka	This study
*P. damone tanais*	BPAL826-11	CCDB-05724_F06	MW186994	Ukraine	Amvrosievka	This study
*P. damone tanais*	BPAL827-11	CCDB-05724_F07	MW186995	Ukraine	Amvrosievka	This study
*P. damone tanais*	BPAL828-11	CCDB-05724_F08	MW186996	Ukraine	Amvrosievka	This study
*P. damone tanais*	KC692328	n/a	KC692328	Russia	Rostov Region, Belaya Kalitva	Vodolazhsky and Stradomsky 2012
*P. icarus*	HM913968	n/a	HM913968	Italy	39.9919°N, 15.7931°E	GenBank
*P. iphigenidesP. iphigenides*	n/a	LOWA422-06	FJ663238	Kyrgyzstan	Transalai Mts, Nura	Lukhtanov et al. 2009
*P. iphigenides iphigenides*	n/a	LOWA423-06	FJ663237	Kyrgyzstan	Transalai Mts, Nura	Lukhtanov et al. 2009
*P. iphigenides iphigenides*	n/a	LOWA424-06	FJ663236	Kyrgyzstan	Transalai Mts, Nura	Lukhtanov et al. 2009
*P. iphigenides iphigenides*	n/a	LOWA514-06	FJ663235	Kyrgyzstan	Alai, Tengizbai Pass	Lukhtanov et al. 2009
*P. iphigenides iphigenides*	n/a	LOWA515-06	FJ663234	Kyrgyzstan	Alai, Tengizbai Pass	Lukhtanov et al. 2009
*P. iphigenides iphigenides*	BPAL1586-12	CCDB-03032_F06	MW194007	Tajikistan	Iskanderkul	This study
*P. iphigenides iphigenides*	BPAL1587-12	CCDB-03032_F07	MW194008	Tajikistan	Iskanderkul	This study
*P. iphigenides iphigenides*	AY496758	n/a	AY496758	Kazakhstan	Shymkent Region, Ugamski Mts	Kandul et al. 2004
*P. iphigenides iphigenides*	AY557155	WE98001	AY557155	Kyrgyzstan	25 km S Song Kul Lake	Wiemers 2003
*P. iphigenides melanius*	BPALB479-18	CCDB-23848_A04	MW186954	Tajikistan	Alai Mts, Jirgatol	This study
* iphigenides melanius *	BPALB480-18	CCDB-23848_A05	MW186955	Tajikistan	Alai Mts, Jirgatol	This study
*P. iphigenides melanius*	BPALB481-18	CCDB-23848_A06	MW186956	Tajikistan	Alai Mts, Jirgatol	This study
*P. iphigenides melanius*	BPALB482-18	CCDB-23848_A07	MW186957	Tajikistan	Alai Mts, Jirgatol	This study
*P. iphigenides melanius*	BPALB483-18	CCDB-23848_A08	MW186958	Tajikistan	Alai Mts, Jirgatol	This study
*P. iphigenides melanius*	BPALB484-18	CCDB-23848_A09	MW186959	Tajikistan	Alai Mts, Jirgatol	This study
*P. iphigenides melanius*	BPALB556-18	CCDB-23848_G09	MW186960	Tajikistan	Peter I Mts, Khorakul Lake	This study
*P. iphigenides melanius*	BPALB558-18	CCDB-23848_G11	MW186961	Tajikistan	Peter I Mts, Mingbulak	This study
*P. iphigenides melanius*	BPALB559-18	CCDB-23848_G12	MW186962	Tajikistan	Peter I Mts, Mingbulak	This study
*P. iphigenides zarmitanus*	BPAL1390-12	CCDB-03030_E12	MW186963	Uzbekistan	Nuratau Mts, Zarmitan	This study
*P. iphigenides zarmitanus*	BPAL1391-12	CCDB-03030_F01	MW186964	Uzbekistan	Nuratau Mts, Zarmitan	This study
*P. iphigenides zarmitanus*	BPAL1392-12	CCDB-03030_F02	MW186965	Uzbekistan	Nuratau Mts, Zarmitan	This study
*P. iphigenides zarmitanus*	BPAL1394-12	CCDB-03030_F04	MW186967	Uzbekistan	Nuratau Mts, Zarmitan	This study
*P. iphigenides zarmitanus*	BPAL1514-12	CCDB-03031_H05	MW186968	Uzbekistan	Hissar Range, Tamshush	This study
*P. iphigenides zarmitanus*	BPAL1515-12	CCDB-03031_H06	MW186969	Uzbekistan	Hissar Range, Tamshush	This study
*P. iphigenides zarmitanus*	BPAL1533-12	CCDB-03032_B01	MW186970	Uzbekistan	Hissar Range, Sangardak	This study
*P. iphigenides zarmitanus*	BPAL1534-12	CCDB-03032_B02	MW186971	Uzbekistan	Hissar Range, Sangardak	This study
*P. iphigenides zarmitanus*	BPAL1535-12	CCDB-03032_B03	MW186972	Uzbekistan	Hissar Range, Sangardak	This study
*P. iphigenides zarmitanus*	BPAL1536-12	CCDB-03032_B04	MW186973	Uzbekistan	Hissar Range, Sangardak	This study
*P. iphigenides zarmitanus*	BPAL1544-12	CCDB-03032_B12	MW186974	Uzbekistan	Hissar Range, Tamshush	This study
*P. iphigenides zarmitanus*	AY556853	DS01001	AY556853	Uzbekistan	Kitabsky reserve	Wiemers 2003
*P. iphigenides zarmitanus* (Holotype)	BPAL1393-12	CCDB-03030_F03	MW186966	Uzbekistan	Nuratau Mts, Zarmitan	This study
*P. juldusus*	BPAL852-11	CCDB-05724_H08	MW186985	Kazakhstan	Almaty Region, Kegen Pass	This study
*P. juldusus*	BPAL870-11	CCDB-05725_B03	MW186986	Kyrgyzstan	Issykkyl, Kadzhisai	This study
*P. juldusus kasachstanus*	AY496759	n/a	AY496759	Kazakhstan	Dzhungarian Alatau	Kandul et al. 2004
*P. juldusus kirgisorum*	BPAL1381-12	CCDB-03030_E03	MW186987	Kyrgyzstan	Shamsi	This study
*P. karatavicus*	BPAL040-10	RPVL-00040	MW186975	Kazakhstan	Karatau Mts, Minzhilgi	This study
*P. karatavicus*	BPAL041-10	RPVL-00041	MW186976	Kazakhstan	Karatau Mts, Minzhilgi	This study
*P. karatavicus*	BPAL042-10	RPVL-00042	MW186977	Kazakhstan	Karatau Mts, Minzhilgi	This study
*P. karatavicus*	BPAL1388-12	CCDB-03030_E10	MW186978	Kazakhstan	Karatau Mts	This study
*P. karatavicus*	AY496760	n/a	AY496760	Kazakhstan	Karatau Mts	Kandul et al. 2004
*P. mediator habievi*	JF343830	ILL087	JF343830	Mongolia	Arshantyn-Nuruu Mts	Vodolazhsky et al. 2011
*P. mediator habievi*	JF343829	ILL086	JF343829	Mongolia	Bayan Ulegei aimak, Elt Gol river	Vodolazhsky et al. 2011
*P. mediator mediator*	EF104602	n/a	EF104602	Mongolia	Altai Mts, Biger	Kandul et al. 2004
*P. phyllides askhabadicus*	BPAL864-11	CCDB-05725_A09	MW186983	Iran	Kuh e Sorkh Mts, Fariman	This study
*P. phyllides askhabadicus*	BPAL865-11	CCDB-05725_A10	MW186984	Iran	Kuh e Sorkh Mts, Fariman	This study
*P. phyllides askhabadicus*	AY954011	n/a	AY954011	Iran	Khorasan, Chakane	Lukhtanov et al. 2005
*P. phyllides kentauensis*	BPAL1382-12	CCDB-03030_E04	MW186980	Kazakhstan	Karatau Mts	This study
*P. phyllides kentauensis*	AY496769	n/a	AY496769	Kazakhstan	Karatau Mts	Kandul et al. 2004
*P. phyllides phyllides*	FJ663239	LOWA633-06	FJ663239	Tajikistan	Iskanderkul	Lukhtanov et al. 2009
*P. phyllides phyllides*	BPAL1328-12	CCDB-03029_H09	MW186979	Uzbekistan	Sairob	This study
*P. phyllides phyllides*	BPAL1578-12	CCDB-03032_E10	MW186981	Tajikistan	Iskanderkul	This study
*P. phyllides phyllides*	BPAL2660-14	CCDB-17967_H11	MW186982	Tajikistan	Sarsaryak	This study
*P. phyllides phyllides*	FJ663240	LOWA571-06	FJ663240	Uzbekistan	Nuratau Mts, Zarmitan	Lukhtanov et al. 2009
*P. phyllides phyllides*	AY496771	n/a	AY496771	Kazakhstan	Karzhantau Mts	Kandul et al. 2004
*P. phyllides phyllides*	AY496770	n/a	AY496770	Kazakhstan	Kirgizski Range	Kandul et al. 2004

Sequences were aligned using the BioEdit software (Hall 1999) and edited manually. Phylogenetic hypotheses were inferred using Bayesian inference as described previously (Vershinina and Lukhtanov 2010; Przybyłowicz et al. 2014; Lukhtanov et al. 2016). Briefly, the Bayesian analysis was performed using the program MrBayes 3.2 (Ronquist et al. 2012) with default settings as suggested by Mesquite (Maddison and Maddison 2015): burn-in = 0.25, nst = 6 (GTR + I + G). Two runs of 10,000,000 generations with four chains (one cold and three heated) were performed. We checked runs for convergence and proper sampling of parameters [effective sample size (ESS) > 200] using the program tracer v1.7.1 (Rambaut et al. 2018). The first 25% of each run was discarded as burn-in. The consensus of the obtained trees was visualized using FigTree 1.3.1 (http://tree.bio.ed.ac.uk/software/figtree/).

### Chromosomal analysis

Karyotypes were studied in 16 adult males representing four species (Table 2) and were processed as previously described (Lukhtanov et al. 2014; Vishnevskaya et al. 2016). Briefly, gonads were removed from the abdomen and placed into freshly prepared fixative (3:1; 96% ethanol and glacial acetic acid) directly after capturing the butterfly in the field. Testes were stored in the fixative for 3–36 months at +4 °C. Then the gonads were stained in 2% acetic orcein for 30–60 days at +18–20 °C. Different stages of male meiosis, including metaphase I (MI) and metaphase II (MII) were examined using an original two-phase method of chromosome analysis (Lukhtanov et al. 2006, 2008). Abbreviation *ca* (circa) means that the count was made with an approximation due to an insufficient quality of preparation or overlapping of some chromosomes or bivalents.

**Table 2.** Chromosome numbers of species of the Polyommatus (Agrodiaetus) damone complex collected by A. Dantchenko (AD), V. Lukhtanov (AV), and Yu. Budashkin and N. Kandul (B & K).

Species	ID number	Chromo-some number	Country	Locality	date	Collector	Reference
*P. damone altaicus*	1987-445	n = ca65	Russia	Altai Mts, Tshulyshman River, 500 m	3–10 August 1987	VL	Lukhtanov 1989
*P. damone altaicus*	1997-1	n = ca65-67	Kazakhstan	near Zaisan city	23 June 1997	VL	Lukhtanov and Dantchenko 2002a
*P. damone altaicus*	1997-2	n = 67	Kazakhstan	Saur Mts, Saikan	2–3 July 1997	VL	Lukhtanov and Dantchenko 2002a
*P. damone damone*	94001	n = 66-67	Russia	Saratov Region, near Volsk	July 1994	AD	Lukhtanov et al. 1997
*P. damone damone*	94002	n = 67	Russia	Saratov Region, near Volsk	July 1994	AD	Lukhtanov et al. 1997
*P. damone damone*	94003	n = 66	Russia	Saratov Region, near Volsk	July 1994	AD	Lukhtanov et al. 1997
*P. damone damone*	94008	n = 67	Russia	Saratov Region, near Volsk	July 1994	AD	Lukhtanov et al. 1997
*P. damone damone*	94010	n = ca66-67	Russia	Saratov Region, near Radishevo	July 1994	AD	This study
*P. damone damone*	95DG5	n = ca67	Russia	South Ural, Kuvandyk	1995	AD	This study
*P. damone damone*	95DG6	n = ca66-67	Russia	South Ural, Kuvandyk	1995	AD	This study
*P. damone irinae*	AD00P077	n = ca67	Russia	Volgograd region	July 2000	AD	Kandul et al. 2007
*P. damone pljushtchi*	95051	n = 65-67	Russia	Crimea, Ai-Petri, 1200 m	14 July 1995	B & K	Kandul 1997
*P. damone pljushtchi*	95054	n = ca66-68	Russia	Crimea, Ai-Petri, 1200 m	14 July 1995	B & K	Kandul 1997
*P. damone pljushtchi*	95055	n = ca65-67	Russia	Crimea, Ai-Petri, 1200 m	14 July 1995	B & K	Kandul 1997
*P. damone pljushtchi*	96009	n = ca65-66	Russia	Crimea, Ai-Petri, 1200 m	10 July 1995	B & K	Kandul 1997
*P. damone pljushtchi*	96010	n = 67	Russia	Crimea, Ai-Petri, 1200 m	10 July 1995	B & K	Kandul 1997
*P. damone pljushtchi*	96011	n = 65	Russia	Crimea, Ai-Petri, 1200 m	10 July 1995	B & K	Kandul 1997
*P. damone pljushtchi*	96012	n = 66-67	Russia	Crimea, Ai-Petri, 1200 m	10 July 1995	B & K	Kandul 1997
*P. damone pljushtchi*	96017	n = ca66-68	Russia	Crimea, Ai-Petri, 1200 m	10 July 1995	B & K	Kandul 1997
*P. damone pljushtchi*	95050	n = 66-67	Russia	Crimea, Ai-Petri, 1200 m	14 July 1995	B & K	Kandul 1997
*P. damone tanais*	95005	n = ca67	Ukraine	Don River basin, Shirokaya balka	26 May 1995	AD	This study
*P. iphigenides iphigenides*	irkeshtam	n = ca66-67	Kyrgyzstan	Transalai Mts (east), Irkeshtam	1996	VL	Lukhtanov and Dantchenko 2002a
*P. iphigenides iphigenides*	1996-4	n = ca66-67	Kyrgyzstan	Naryn Region, Chaek	4 July 1996	VL	This study
*P. iphigenides iphigenides*	1996-3	n = ca66	Kyrgyzstan	Moldatoo Mts, Teke-Uyuk	30 June 1996	VL	This study
*P. iphigenides iphigenides*	1995 – Chiitala	n = 67	Kyrgyzstan	Alai Mts, Chiitala village, 2300 m	1995	VL	Lukhtanov and Dantchenko 2002a
*P. iphigenides iphigenides*	1994-1	n = ca66-67	Tajikistan	Iskanderkul	July 1994	VL	This study
*P. iphigenides iphigenides*	95205	n = ca66-67	Kyrgyzstan	Alai Mts, Chiitala village, 2300 m	1995	VL	Lukhtanov and Dantchenko 2002a
*P. iphigenides iphigenides*	NK00P823 AY496758	n = ca65-67	Kazakhstan	Ugamski range	June 2000	VL	Lukhtanov et al. 2005
*P. iphigenides melanius*	068K18A	n = 66	Tajikaistan	Alai Mts, Jirgatol	July 2018	AD	This study
*P. iphigenides melanius*	077K18A	n = 67	Tajikaistan	Alai Mts, Jirgatol	July 2018	AD	This study
*P. iphigenides melanius*	Tj002	n = 66	Tajikaistan	Peter I Mts	July 20218	VL	This study
*P. iphigenides zarmitanus*	94L01	n = ca66-68	Uzbekistan	Nuratau Mts, Zarmitan, 1300 m	11–13 June 1994	VL	This study
*P. iphigenides zarmitanus*	94L03	n = ca68	Uzbekistan	Nuratau Mts, Zarmitan, 1300 m	11–13 June 1994	VL	This study
*P. iphigenides zarmitanus*	94L04	n = 67	Uzbekistan	Nuratau Mts, Zarmitan, 1300 m	11–13 June 1994	VL	This study
*P. iphigenides zarmitanus*	94L54	n = ca66-67	Uzbekistan	Hissar Range, Sangardak, 1600 n	2 July 1994	VL	Lukhtanov and Dantchenko 2002a
*P. iphigenides zarmitanus*	94L61	n = 67	Uzbekistan	Hissar Range, Tamshush, 1800 n	5–7 July 1994	VL	Lukhtanov and Dantchenko 2002a
*P. iphigenides zarmitanus*	94L64	n = 66	Uzbekistan	Hissar Range, Tamshush, 1800 n	5–7 July 1994	VL	Lukhtanov and Dantchenko 2002a
*P. iphigenides zarmitanus*	94L74	n = ca65-67	Uzbekistan	Samarkand Region, Aman-Kutan	7 July 1994	VL	Lukhtanov and Dantchenko 2002a
*P. iphigenides zarmitanus*	94L75	n = ca65-67	Uzbekistan	Samarkand Region, Aman-Kutan	7 July 1994	VL	Lukhtanov and Dantchenko 2002a
*P. iphigenides zarmitanus*	1994-2	n = 67	Uzbekistan	Nuratau Mts, Zarmitan, 1300 m	11–13 June 1994	VL	This study
*P. iphigenides zarmitanus*	1994-3	n = 67	Uzbekistan	Tamshush	1994	VL	Lukhtanov and Dantchenko 2002a
*P. iphigenides zarmitanus*	1994-4	n = 67	Uzbekistan	Samarkand Region, Aman-Kutan	7 July 1994	VL	Lukhtanov and Dantchenko 2002a
*P. juldusus kasachstanus*	1997-3	n = 67	Kazakhstan	Dzhungarian Alatau, Kysylagash	June 2000	VL	Lukhtanov and Dantchenko 2002a
*P. karatavicus*	2000-K	n = 67	Kazakhstan	Karatau Mts, Minzhilgi	June 2000	VL	Lukhtanov et al. 2005
*P. phyllides askhabadicus*	F456	n = ca66-67	Iran	Kuh-e-Sorkh Mts., Torbat-e-Heydariyeh	7 July 2003	VL&D	This study
*P. phyllides askhabadicus*	VL03F523 AY954011	n = 67	Iran	Khorasan, Chakane	9 July 2003	VL&D	This study
*P. phyllides phyllides*	95204	n = ca65-67	Kyrgyzstan	Naryn Region, Moldatoo Mts, Chon-Konduk	26 July 1995	VL	This study
*P. phyllides phyllides*	NK00P672 AY496770	n = ca66-67	Kazakhstan	Kazakhstan, Kirgizski range,	June 2000	VL	Lukhtanov and Dantchenko 2002a
*P. phyllides phyllides*	NK00P808 AY496771	n = ca66-67	Kazakhstan	Kazakhstan, Karzhantau mts	June 2000	VL	Lukhtanov and Dantchenko 2002a

Leica DM2500 light microscope equipped with HC PL APO 100×/1.44 Oil CORR CS lens and S1/1.4 oil condenser head was used for bright-field microscopy analysis. Leica DM2500 light microscope equipped with HC PL APO 100×/1.40 OIL PH3 lens was used for phase-contrast microscopy analysis.

## Results

### DNA-barcode analysis

**Figure 1.**
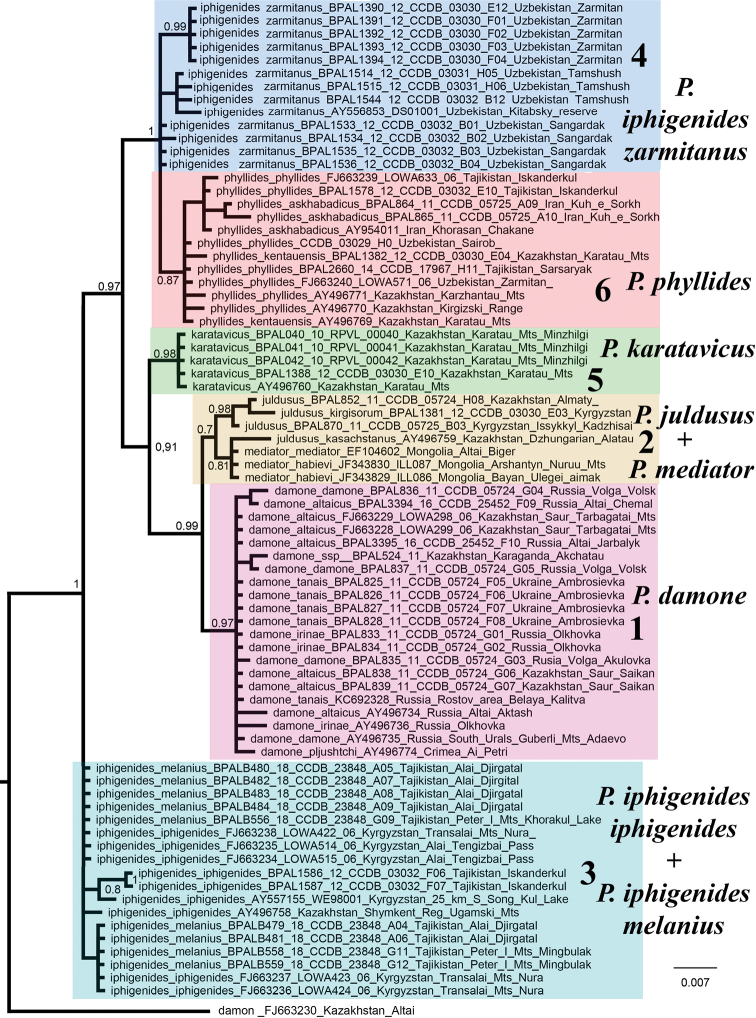
The Bayesian majority rule consensus tree of the analyzed samples of Polyommatus (Agrodiaetus) inferred from *COI* sequences. *Polyommatus icarus* is used to root the tree (not shown). Species and subspecies names, GenBank accession numbers, museum ID numbers and localities are shown to the right of the branches. Bayesian posterior probabilities higher than 0.5 are shown next to the recovered branches. 1–6 are clusters (see explanation in the text).

Phylogenetic analysis revealed six clusters of closely related individuals within the P. (A.) damone species complex (Fig. 1). Of these clusters, four groups were monophyletic and two groups were paraphyletic. The lineages of P. (A.) damone (I) and P. (A.) karatavicus Lukhtanov, 1990 (V) were highly supported. The lineage of P. (A.) phyllides (Staudinger, 1886) (VI) and the lineage [(P. (A.) mediator Dantchenko et Churkin, 2003 + P. (A.) juldusus kasachstanus Lukhtanov et Dantchenko, 1994)] (II) had medium support. The clusters III [P. (A.) iphigenides iphigenides (Staudinger, 1886) + P. (A.) iphigenides melanius (Staudinger, 1886)] and VI (P. (A.) iphigenides zarmitanus subsp. nov.) appeared on the tree as two distinct, not closely related paraphyletic taxa.

### Chromosomal analysis

**Figure 2.**
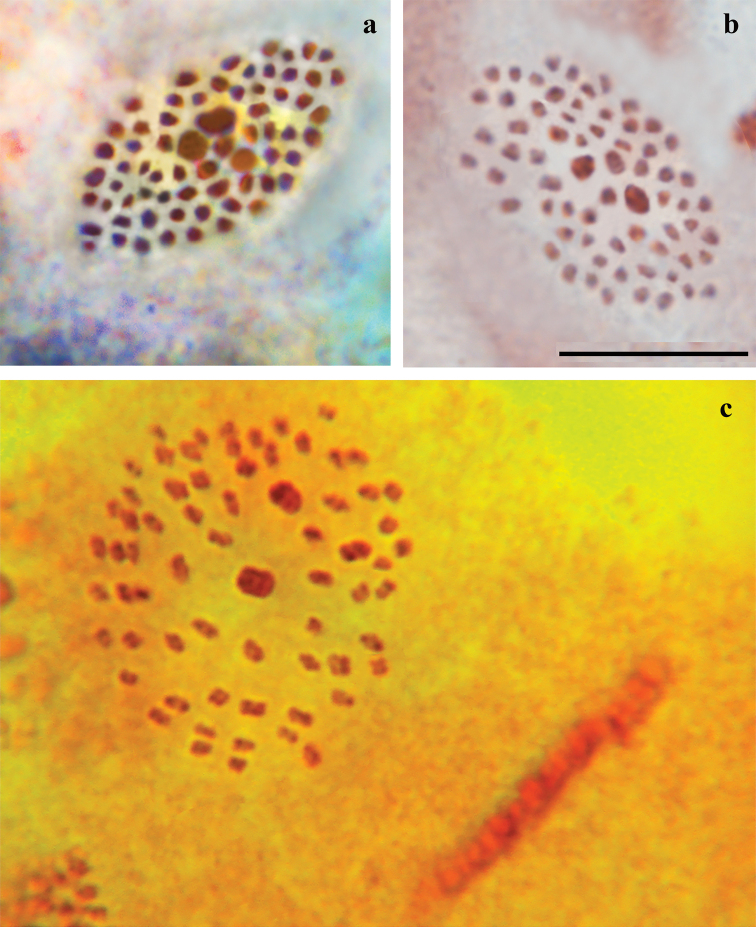
Karyotypes of Polyommatus (Agrodiaetus) iphigenides melanius and P. (A.) phyllides askhabadicus**a**P. (A.) iphigenides melanius, sample 077K18A, MI, n = 67, phase-contrast **b**P. (A.) iphigenides melanius, sample 068K18A, MI, n = 66 **c**P. (A.) phyllides askhabadicus, sample F523, MI, n = 67. Scale Bar: 10 μm.

Chromosomal analysis of three males of P. (A.) damone damone, of a single male of P. (A.) damone tanais Dantchenko et Pljushtch, 1993, of two males of P. (A.) iphigenides iphigenides, of three males of P. (A.) iphigenides melanius, of a single male of P. (A.) phyllides phyllides, of two males of P. (A.) phyllides askhabadicus (Forster, 1960) and four males of P. (A.) iphigenides zarmitanus subsp. nov. revealed the same (or almost the same) haploid chromosome number n = 66 or n = 67 in all studied taxa (Table 2). The karyotype structure was also found to be identical in all studied individuals, with three large bivalents in the center of metaphase plates (Fig. 2). Bivalent 1 was 1.2–1.5 times larger than bivalent 2, and the latter was 1.2–1.5 times larger than bivalent 3.

## Discussion

### Chromosomal stasis

It has been found that all taxa within P. (A.) damone species complex demonstrate chromosomal stasis and share the same or very similar haploid chromosomal number (n = 66 or n = 67). This result is unexpected since the karyotypes are known to be very diverse and species-specific in the subgenus Agrodiaetus.

It is believed that an unusual diversity of karyotypes is the most remarkable characteristic of *Agrodiaetus*. Species of this subgenus exhibit one of the highest ranges in chromosome numbers in the animal kingdom (Vershinina and Lukhtanov 2017). In *Agrodiaetus* haploid chromosome numbers (n) range from n = 10 in P. (A.) caeruleus (Staudinger, 1871) to n = 134 in P. (A.) shahrami (Skala, 2001) (Lukhtanov et al. 2005). The genus *Polyommatus* as a whole shows numbers from n = 10 to n = 226 (Lukhtanov 2015). Additionally, the subgenus Agrodiaetus demonstrates a high level of karyotypic differentiation with respect to chromosome size (Lukhtanov and Dantchenko 2002b) and variation in number of chromosomes bearing ribosomal DNA clusters (Vershinina et al. 2015). These differences provide reliable characters for species delimitation, description and identification (de Lesse 1960, 1963; Lukhtanov and Dantchenko 2002a, b).

The P. (A.) damone species complex represents an exception. In this group divergence in several phylogenetic lineages was not accompanied by changes in karyotypes, and the chromosome number n = 66-67 is the synapomorphic character for the species of the group.

### DNA-barcode clusters

**Figure 3.**
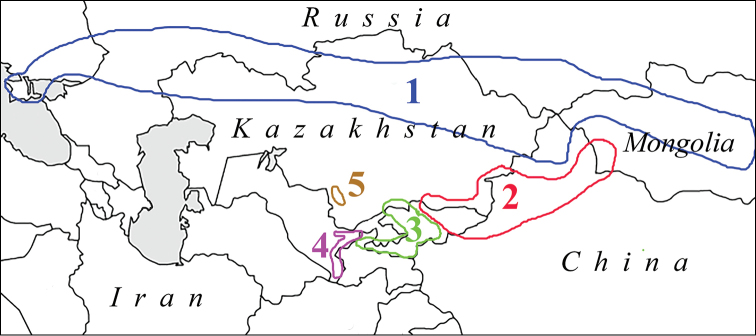
Distribution areas of the *COI* clusters revealed in this study. Cluster 1 corresponds to *P. damone*. Cluster 2 corresponds to *P. juldusus* + *P. mediator*. Cluster 3 corresponds to *P. iphigenides* (including *P. iphigenides melanius*). Cluster 4 corresponds to *P. zarmitanus*. Cluster 5 corresponds to *P. karatavicus*.

**Figure 4.**
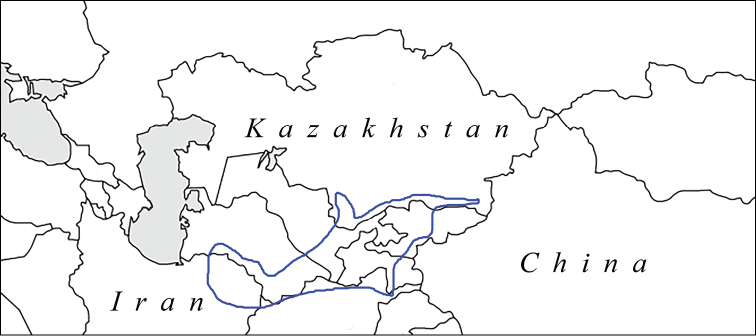
Distribution area of *P. phyllides* (cluster 6).

The DNA-barcode clusters revealed in our study correspond well to traditionally recognized species and certain specific geographic areas (Figs 3, 4). Cluster 1 includes specimens from the Crimea in the west to Altai and Saur-Tarbagatai Mts in the east and corresponds to P. (A.) damone. Cluster 2 includes specimens from NE Kyrgyzstan, SE Kazakhstan and SW Mongolia and corresponds to P. (A.) juldusus + P. (A.) mediator. Cluster 3 includes specimens from western and southern Kyrgyzstan, southern Kazakhstan and Tajikistan and corresponds to P. (A.) iphigenides iphigenides + P. (A.) iphigenides melanius. Cluster 4 includes specimens from West Hissar in Uzbekistan and western Tajikistan and corresponds to P. (A.) iphigenides zarmitanus subsp. nov. which will be described below. Cluster 5 includes specimens from Karatau Mts in Kazakhstan and corresponds to P. (A.) karatavicus. Cluster 6 (Fig. 4) includes specimens from northeastern Iran to southeastern Kazakhstan and corresponds to P. (A.) phyllides.

Cluster 6 (=*P. phyllides*) is sympatric with cluster 2 (=*P. juldusus*) in northern Kyrgyzstan and southeastern Kazakstan, with cluster 3 (=*P. iphigenides iphigenides*+*P. iphigenides melanius*) in Kyrgyzstan and Tajikistan, with cluster 4 (=*P. iphigenides zarmitanus*) in Uzbekistan and western Tajikistan, with cluster 5 (=*P. karatavicus*) in Karatau Mts in Kazakhstan (Eckweiler and Bozano 2016; our personal observations).

### Taxonomic interpretations

#### Clusters 1 (*P. damone*), 2 (*P. juldusus* + *P. mediator*) and 5 (*P. karatavicus*)

We follow previous research (Dantchenko 2000; Dantchenko and Churkin 2003, Lukhtanov et al. 2005) in interpreting clusters 1 (*P. damone*), 2 (*P. juldusus* and *P. mediator*) and 5 (*P. karatavicus*) (see Taxonomic conclusions below). P. (A.) mediator was described as a species which is intermediate between P. (A.) damone and P. (A.) juldusus, but more similar to P. (A.) juldusus due to specific white pubescence of the costal area of the forewings (Dantchenko and Churkin 2003). This conclusion is now supported by molecular data: on the phylogenetic tree it appears as a clade, which also includes P. (A.) juldusus kasachstanus, and as a sister clade to P. (A.) juldusus juldusus + *P. (A.) juldususkirgisorum.*

Up to our knowledge there are no data on sympatry of P. (A.) mediator and P. (A.) damone in Mongolia as it was reported or supposed earlier (Bálint and Johnson 1987; Bálint 1989).

#### Cluster 3 (*P. iphigenides iphigenides* + *P. iphigenides melanius*)

Polyommatus (Agrodiaetus) iphigenides is highly polymorphic with regard to the black suffusion on the wing upperside and the marginal and submarginal part of the wing underside in males as well as the white streak on hindwings in both sexes. In extreme cases, the suffusion can be practically absent resembling the upperside in *P. damone* or may extend almost to the discal spot which is observed as a fixed feature in two other taxa, *P. iphigenides melanius* and *P. juldusus kirgisorum*. The white streak is also very variable from clear visibility to complete absence. The taxa P. (A.) samusi Korb, 2017 (syn. nov.) and P. (A.) melanius komarovi Korb, 2017 (syn. nov.) are mainly described on the base of such extreme forms of the same population. Therefore, we consider these taxa as junior subjective synonyms of *P. (A.) iphigenidesiphigenides.*

Cluster 3 also includes the taxon described as *Lycaena kindermanni* var. *Melania* Staudinger, 1886. For a long time, due to lack of material it had been considered to be a melanized form of P. (A.) iphigenides iphigenides (e.g. Forster 1960). But in recent years it has been treated as a separate species P. (A.) melanius with a local, nearly dot-like distribution in the border area between southwestern Kyrgyzstan and eastern Tajikistan in the Kyzylsu/Surkhob River basin (Dantchenko 2000; Eckweiler and Bozano 2016). We found that DNA barcodes of P. (A.) iphigenides and P. (A.) melanius are identical or differ by non-fixed 1–2 nucleotide substitutions. The main feature of P. (A.) melanius, a wide dark marginal border on the fore- and hindwings, is quite stable for the diagnosis of the taxon; however, the tendency towards such a wide border is expressed in different populations of P. (A.) iphigenides, too. Therefore, this trait can be hardly considered a species-specific character. Here we argue that P. (A.) melanius is rather a subspecies P. (A.) iphigenides than a species. However, this is not a final conclusion. There is indirect evidence in favour of a possible species status of P. (A.) melanius, e.g. the distribution areas of P. (A.) iphigenides iphigenides and P. (A.) iphigenides melanius almost touch each other, and an intergradation zone would be expected between them. However, such a zone is still unknown, and specimens of P. (A.) iphigenides iphigenides and P. (A.) iphigenides melanius from very close localities are clearly differentiated. We suppose that genome-wide analysis may be useful to verify the taxonomic status of P. (A.) iphigenides melanius.

#### Cluster 4 (*P. iphigenides zarmitanus*)

Morphologically this group is close to *P. ipigenides iphigenides*, whereas with regard to mitochondrial DNA it is close to sympatric species *P. phyllides* which is morphologically very different. In our opinion, two alternative evolutionary scenarios can explain this pattern.

##### Scenario 1

The cluster 4 (*P. iphigenides zarmitanus*) and the lineage 6 (*P. phyllides*) are sister species which recently evolved from a common ancestor by means of sympatric speciation.

##### Scenario 2

Cluster 3 (*P. iphigenides*) and cluster 4 (*P. iphigenides zarmitanus*) are sister taxa evolved in allopatry; therefore, they share an ancestral type of the wing pattern and coloration, although differentiated with respect to DNA barcodes. The similarity between completely sympatric cluster 4 (*P. iphigenides zarmitanus*) and lineage 6 (*P. phyllides*) is a result of ancient mitochondrial introgression.

Analysis of multiple nuclear markers is required in order to distinguish between these two scenarios. Scenario 2 seems to be more probable since mitochondrial introgression is not a rare phenomenon in butterflies (e.g. Gompert 2008; Cong et al. 2017) and is also documented in the subgenus Polyommatus (Agrodiaetus) (Lukhtanov et al. 2015). Therefore, below we describe the new lineage discovered in West Hissar region as a subspecies of *P. iphigenides*.

#### Cluster 6 (*P. phyllides*)

There is no doubt that the cluster 6 (*P. phyllides*) is a distinct species, since it is a monophyletic lineage (Fig. 1), which is morphologically and ecologically differentiated (Dantchenko 2000, Eckweiler and Bozano 2016) and sympatric with P. (A.) iphigenides iphigenides, P. (A.) iphigenides melanius, P. (A.) iphigenides zarmitanus, P. (A.) karatavicus and P. (A.) juldusus.

### New subspecies description

#### Polyommatus (Agrodiaetus) iphigenideszarmitanus subsp. nov.

Taxon classificationAnimaliaLepidopteraLycaenidae

6CC57375-07BE-5143-90D4-4C64ED698ADC

http://zoobank.org/092F10F6-B5E7-46C3-AA26-3A4D82F1F6D7

##### Holotype.

**Figure 5.**
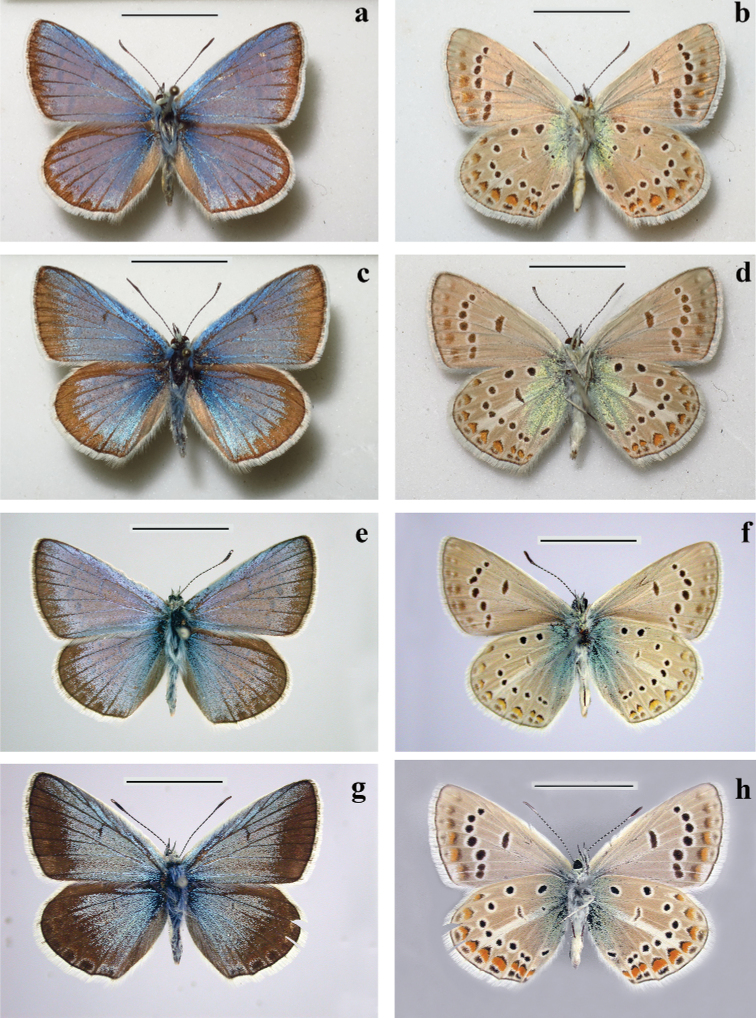
Males of Polyommatus (Agrodiaetus) iphigenides**a, b** upperside (**a**) and underside (**b**) of the holotype of P. (A.) iphigenides zarmitanus subsp. nov. **c, d** upperside (**c**) and underside (**d**) of P. (A.) iphigenides iphigenides, Tajikistan, Transalai Mts, Shibe village **e, f** upperside (**e**) and underside (**f**) of the Lectotype of P. (A.) iphigenides iphigenides, “Namangan”, in Museum für Naturkunde, Humboldt-Universität zu Berlin **g, h** upperside (**g**) and underside (**h**) of the Lectotype of P. (A.) iphigenides melanius, in Museum für Naturkunde, Humboldt-Universität zu Berlin. Scale Bars: 10 mm.

(Fig. 5a, b), male, BOLD process ID BPAL1393-12, field # CCDB-03030_F03, GenBank accession number MW186966; Uzbekistan, Samarqand Region, Nuratau Mts, near Zarmitan village, 40.40°N, 66.69°E, 1300 m, 11–13 June 1994, V. Lukhtanov leg., deposited in the Zoological Institute of the Russian Academy of Science (St. Petersburg).

##### *COI* barcode sequence of the holotype.

ACATTATATTTTATTTTTGGAATTTGAGCAGGAATAGTAGGGACATCCCTAAGAATTTTAATCCGTATAGAATTGAGAACT CCTGGATCCTTAATTGGAGACGATCAAATTTATAATACTATTGTTACAGCCCATGCATTTATTATAATTTTTTTTATAGTTA TACCTATTATAATTGGGGGATTTGGTAATTGATTAGTTCCTTTAATATTAGGAGCACCTGATATAGCCTTCCCCCGATTAAA TAATATAAGATTCTGATTATTACCGCCATCATTAATACTACTAATTTCCAGAAGAATTGTAGAAAATGGAGCAGGAACAGGA TGAACAGTTTACCCCCCACTTTCATCTAATATTGCACATAGAGGATCATCTGTAGATTTAGCAATTTTCTCTCTTCATTTAG CAGGAATTTCTTCAATTTTAGGAGCAATTAATTTTATTACAACTATTATTAACATACGGGTAAATAATTTATCATTTGATCA AATATCATTATTTATTTGAGCAGTAGGAATTACAGCATTATTATTACTTTTATCTTTACCTGTATTAGCTGGAGCAATTACC ATATTATTAACAGATCGAAACCTTAATACCTCATTCTTTGACCCAGCTGGTGGGGGAGATCCAATTTTATATCAACATTTA.

##### Paratypes.

39 males, 14 females: Uzbekistan, Samarqand Region, Nuratau Mts, near Zarmitan village, 40.40°N, 66.69°E, 1300 m, 11–13 June 1994, V. Lukhtanov leg. 2 males: Uzbekistan, Qashqadaryo Region (old spelling: Kashkadarya Region), Hissar Range (west), near Tamshush village, 38.98°N, 67.35°E, 1800 m, 18–20 June 1994, V. Lukhtanov leg. 20 males: Uzbekistan, Qashqadaryo Region (old spelling: Kashkadarya Region), Hissar Range (west), near Tamshush village, 38.98°N, 67.35°E, 1800 m, 5–7 July 1994, V. Lukhtanov leg. 11 males, 2 females: Uzbekistan, Surxondaryo Region (old spelling: Surkhandarya Region), Hissar Range (west), Sangardak, 38.55°N, 67.50°E, 1600 m, 2 July 1994, V. Lukhtanov leg. 60 males, 21 females: Uzbekistan, Samarqand Region, Zeravshansky Range, Aman-Kutan, 1800 m, 39.27°N, 66.90°E, 7 July 1994, V. Lukhtanov leg. 13 males: Tajikistan, Sughd Region, Zeravshansky Range, Padzhrud village, 39.37°N, 68.03°E, 1300 m, 17 males, 13 males, 10 June 1994. All above paratypes are deposited in the Zoological Institute of the Russian Academy of Science (St. Petersburg). 5 males: Uzbekistan, [Jizzakh region], Usmat vic., 1700 m, 30.06.1988, V. Tshikolovets leg., in State Darwin Museum, Moscow. 15 males: [Uzbekistan], Aman-Kutan near Samarqand, 20 June 1938, A. Tsvetaev leg., in State Darwin Museum, Moscow. 26 males, 1 female: [Tajikistan], Hisar-Alai, Zeravshansky Range, Farob, 2000 m, 4 July 1998, G.D. Samodurov leg., in State Darwin Museum, Moscow. 1 female: Tajikistan, West Hissar, Nofin lake, 2400, 17 July 1993, S. Churkin leg., in State Darwin Museum, Moscow. 32 males: [Uzbekistan], Aman-Kutan near Samarqand, 15–25 June 1938, A. Tsvetaev leg., in Zoological Museum Moscow University, Moscow (ZMMU). 7 males: [Uzbekistan], Aman-Kutan near Samarqand, 20–23 June 1938, G.Pashin leg., in ZMMU. 2 males: [Uzbekistan], Aman-Kutan near Samarqand, 27 July and 5 August 1937, A. G. Pashin leg., in ZMMU. 3 females: [Uzbekistan], Aman-Kutan near Samarqand, 15–26 June 1938, A. Tsvetaev leg., in ZMMU. 8 males: Tajikistan, West Hissar, Khazorchashma lake, 2800, 26 July 1993, S. Churkin leg.; 1 female: Tajikistan, West Hissar, Nofin lake, 2400, 17 July 1993, S. Churkin leg., in coll. Churkin (Reutov, Russia). 2 males, 1 female: Uzbekistan, West Hissar, Boysun Mts, Mochay, 1500 m, 26 June 1980, V. Tuzov leg., in coll. Tuzov (Moscow). 10 males, 1 female: [Uzbekistan], Aman-Kutan near Samarqand, 19–23 June 1938, A. Tsvetaev leg., in coll. Sochivko A. (Moscow). 10 males, 1 female: [Tajikistan], Hissar-Alai, Zeravshansky Range, Farob, 2000 m, 4 July 1998, L. Nikolaevsky leg., in coll. V. Kalinin, Moscow.

##### Description.

**Males. *Forewing*** length 15–17 mm.

***Upperside***: Ground color bright glossy milky blue with narrow black marginal line, marginal part of forewings and hindwings dusted with black scales, discal strokes may be present or absent, veins darkened, costal area of the forewings white, hindwings with antemarginal spots, fringe white.

***Underside***: Forewing ground color light grey, submarginal row blurred, but clear visible; discoidal strokes black, bordered with white; postdiscal rows of black spots bordered with white, basal black spots absent; hindwing ground color light grey, basal area with strong greenish blue suffusion between wing root and basal spots; basal spots small, bordered with white, discal stroke less prominent than on forewings; postdiscal row of black spots bordered with white, submarginal and antemarginal marking strong and clear visible; submarginal row bordered distally with reddish lunules, more pronounced to anal end of row; white streak not contrasting, often hardly noticeable or absent at all, fringes pale grayish.

**Genitalia.** The male genitalia have a structure typical for other species of the subgenus Agrodiaetus (Coutsis 1986, Eckweiler and Bozano 2016).

**Figure 6.**
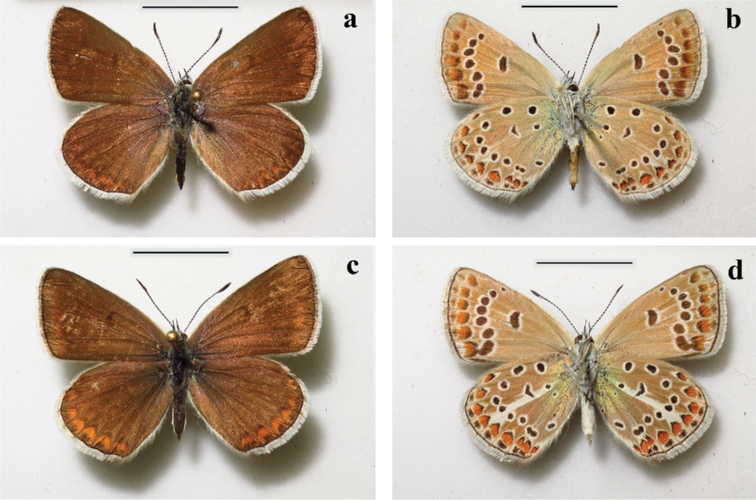
Females of Polyommatus (Agrodiaetus) iphigenides**a, b** upperside (**a**) and underside (**b**) of the paratype of P. (A.) iphigenides zarmitanus subsp. nov. **c, d** upperside (**c**) and underside (**d**) of P. (A.) iphigenides iphigenides, Tajikistan, Transalai Mts, Shibe village. Scale Bars: 10 mm.

**Females.** (Fig. 6a, b) ***Forewing*** length 15–17 mm.

***Upperside***: Ground color brown with slightly darker veins, discal strokes present, submarginal and antemarginal marking almost absent on fore wings and strong and clear visible on hindwings, antemarginal black spots on hindwings bordered with orange lunules, fringe whitish.

***Underside***: ground color and general design as in males but darker, brownish grey, greenish blue basal suffusion near invisible, white streak on hindwings clear visible, enlarged distally, fringe light greyish.

##### Diagnosis.

The new subspecies is distinguished phenotypically from the most similar *P. iphigenides iphigenides* (Figs 5c–f, 6c, d) by the underside of the hind wing, which has a paler and less contrasting coloration. The white streak is also dim and weakly stands out against the background of the wing, is often reduced or absent. The same can be said about the basal greenish-blue suffusion: it is dim and weakly stands out against the background of the wing; its size, on average, is much smaller than that in *P. iphigenides iphigenides*. As a rule, it is limited by black dots of the basal row, while in *P. iphigenides iphigenides* it usually extends further in the distal direction, sometimes to spots of the discal row. This suffusion itself has a more greenish tint than that in *P. iphigenides iphigenides* (in the latter, it is more blue). The new species always has black dots of the basal row (although they are small), while in another species they are reduced.

The main differences between the species are still in the molecular characters. *Polyommatus iphigenides zarmitanus* can be distinguished from *P. iphigenides iphigenides* by using molecular markers from the *COI* gene. These mitochondrial diagnostic characters are in the following positions in the *COI* barcode region: adenine (A) in position 22, cytosine (C) in position 132, guanine (G) in position 180, cytosine (C) in position 286, guanine (G) in position 468, guanine (G) in position 468, and guanine (G) in position 627.

The new subspecies differs from sympatric (syntopic and synchronous) *P. phyllides* by milky blue (not greenish blue) wing upperside and white pubescence of the costal area of the forewings in males and by light grey color of the wing underside (*P. phyllides* has specific warm pinkish grey color of the wing underside). It also differs from *P. phyllides* by diagnostic nucleotide guanine (G) in position 627 of the *COI* barcode region.

##### Distribution area

**Figure 7.**
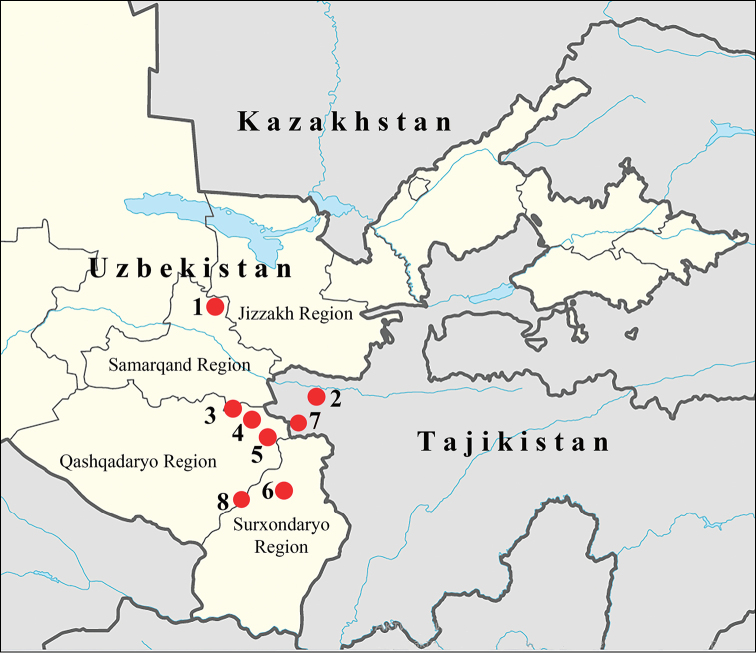
Distribution area of P. (A.) iphigenides zarmitanus. 1 is the type-locality, Zarmitan in Nuratau Mts. 2 is Padzhrud village in Zeravshansky Range. 3 is Aman-Kutan near Samarqand. 4 is Kitabsky Reserve in Hissar Range. 5 is Tamshush in Hissar Range. 6 is Sangardak in Hissar Range. 7 are Khazorchashma and Nofin lakes in Hissar Range. 8 is Mochay in Boysun Mts.

**(Fig. 7).** Uzbekistan: West part of the Hissar Range, Zeravshan Mts, Nuratau Mts, Boysun (= Baisuntau) Mts. Tajikistan: west part of the Zeravshan valley and Zeravshansky Range, West Hisar Range.

##### Habitat and biology.

Stony steppe and dry meadows from 1200 up to 2800 m alt. Flight period from late May to first decade of August in a single generation. The new subspecies flies syntopically and synchronously with the second generation of P. (A.) phyllides, but on average about one decade earlier. Host plant is preliminary determined as *Hedysarum* sp. (Fabaceae).

##### Etymology.

The name *zarmitanus* is an adjective of the masculine gender. This name originates from Zarmitan, the village in Uzbekistan.

### Taxonomic conclusion

The discovered topology (Fig. 1) can be considered as a signal to taxonomic rearrangement within the group. However, since the volume of the studied material of these taxa is small, we prefer to leave the existing taxonomic hypotheses. Additionally, we assume that the hypothesis of the existence of a species called P. (A.). *altaiensis* with subspecies P. (A.) altaiensis altaiensis, P. (A.) altaiensis bogdoolensis Dantchenko et Lukhtanov, 1997, P. (A.). *altaiensismediator* and P. (A.) altaiensis habievi Yakovlev, 2004 (Eckweiler and Bozano 2016) is speculative and not supported by significant morphological characters.

Based on the stated above, we propose the following taxonomic arrangement of the *P. damone* species complex:

**P. (A.) damone (Eversmann, 1841)**

P. (A.) damone pljushtchi (Lukhtanov et Budashkin, 1993)

P. (A.) damone tanais Dantchenko et Pljushtch, 1993

P. (A.) damone irinae Dantchenko, 1997

P. (A.) damone damone (Eversmann, 1841)

P. (A.) damone altaicus (Elwes, 1899) (= Lycaena damone var. sibirica Staudinger, 1899; = *Agrodiaetus carmon altaiensis* Forster, 1956)

P. (A.) damone walteri Dantchenko et Lukhtanov, 1993

P. (A.) damone bogdoolensis Dantchenko et Lukhtanov, 1997

P. (A.) damone fabiani Bálint, 1997

**P. (A.) mediator Dantchenko et Churkin, 2003** (= *Agrodiaetus mediator habievi* Yakovlev, 2004)

**P. (A.) juldusus (Staudinger, 1886)** (= Lycaena damone var. duplicata A. Bang-Haas, 1910)

P. (A.) juldusus juldusus (Staudinger, 1886)

P. (A.) juldusus kirgisorum Lukhtanov et Dantchenko, 1994 (=*P. hyrsyz* Koçak et Kemal, 2001; = *P. kirgisorum khamul* Korb, 2009; = *P. kirgisorum gorthaur* Korb, 2009)

P. (A.) juldusus kasachstanus Lukhtanov et Dantchenko, 1994

P. (A.) juldusus rueckbeili Forster, 1960

P. (A.) juldusus tianchinensis Eckweiler, 2013

**P. (A.) iphigenides (Staudinger, 1886)**

P. (A.) iphigenides iphigenides (Staudinger, 1886) (= *P. ishkashimicus alajanus* Korb, 1997; = *P. samusi* Korb, 2017, syn. nov.; = *P. melanius komarovi* Korb, 2017, syn. nov.)

P. (A.) iphigenides melanius (Staudinger, 1886)

P. (A.) iphigenides zarmitanus subsp. nov.

**P. (A.) karatavicus Lukhtanov, 1990**

**P. (A.) phyllides (Staudinger, 1886)**

P. (A.) phyllides phyllides (Staudinger, 1886)

P. (A.) phyllides askhabadicus (Forster, 1960)

P. (A.) phyllides kentauensis Lukhtanov, 1990

P. (A.) phyllides urumbash Churkin et Zhdanko, 2008

## Supplementary Material

XML Treatment for Polyommatus (Agrodiaetus) iphigenideszarmitanus

## References

[B1] Bálint Z (1989) Recently collected lycaenid butterflies of Mongolia (V) (Lep., Lycaenidae). Galathea 5: 101–112.

[B2] Bálint Z Johnson K (1987) Reformation of the Polyommatus section with taxonomic and biogeographic overview (Lepidoptera, Lycaenidae, Polyommatini). Neue Entomologische Nachrichten 40: 1–68.

[B3] Cong Q Shen J Borek D Robbins RK Opler PA Otwinowski Z Grishin NV (2017) When *COI* barcodes deceive: complete genomes reveal introgression in hairstreaks. Proceedings of the Royal Society B – Biological Sciences 284: 20161735. 10.1098/rspb.2016.1735 PMC5310595 28179510

[B4] Coutsis JG (1986) The blue butterflies of the genus *Agrodiaetus* Hübner (Lepidoptera, Lycaenidae): symptoms of taxonomic confusion. Nota Lepidopterologica 9: 159–169.

[B5] Dantchenko AV (1997) Notes on the biology and distribution of the *damone* and *damocles* species-complexes of the subgenus Polyommatus (Agrodiaetus) (Lepidoptera: Lycaenidae). Nachrichten des Entomologischen Vereins Apollo 16: 23–42.

[B6] Dantchenko AV (2000) Genus *Agrodiaetus*. In: Tuzov VK (Ed.) Guide to the butterflies of Russia and adjacent territories, Vol.2. Sofia: Moscow, Pensoft, 196–214.

[B7] Dantchenko A Churkin S (2003) A new species of the genus *Polyommatus* Latreille, 1804 from Mongolia (Lepidoptera, Lycaenidae). Neue Entomologische Nachrichten 54: 5–13.

[B8] Dantchenko A Lukhtanov VA (1993) Zur Systematik und Verbreitung der Arten der Polyommatus (Agrodiaetus) damone-Gruppe (Lepidoptera, Lycaenidae) Südosteuropas und Südwestsibiriens. Atalanta 24(1/2): 75–83, 324–325.

[B9] de Lesse H (1960) Speciation et variation chromosomique chez les Lepidopteras Rhopaloceras. Annales des Sciences Naturelles Zoologie et Biologie Animale, 12e série 2: 1–223.

[B10] de Lesse H (1963) Variation chromosomique chez *Agrodiaetus*carmon H. S. et *A. cyanea* Stgr. Revue Française d’Entomologie 30: 177–181.

[B11] deWaard JR Ivanova NV Hajibabaei M Hebert PDN (2008) Assembling DNA barcodes: analytical protocols. In: Martin CC (Ed.) Environmental Genomics, Methods in Molecular Biology. Humana Press, Totowa, New Jersey, 410: 275‒283. 10.1007/978-1-59745-548-0_15 18642605

[B12] Eckweiler W Bozano GC (2016) Guide to the butterflies of the Palearctic region. Lycaenidae part IV. Milano, Omnes artes, 132 pp.

[B13] Forster W (1956) Bausteine zur Kenntnis der Gattung *Agrodiaetus* Scudd. (Lep. Lycaen) I. Zeitschrift der Wiener Entomologischen Gesellschaft 41: 42–61, 70–89, 118–127.

[B14] Forster W (1960) Bausteine zur Kenntnis der Gattung *Agrodiaetus* Scudd. (Lep. Lycaen) II. Zeitschrift der Wiener Entomologischen Gesellschaft 45: 105–142.

[B15] Gompert Z Forister ML Fordyce JA Nice CC (2008) Widespread mito‐nuclear discordance with evidence for introgressive hybridization and selective sweeps in *Lycaeides*. Molecular Ecology 17: 5231–5244. 10.1111/j.1365-294X.2008.03988.x 19120997

[B16] Hajibabaei M deWaard JR Ivanova NV Ratnasingham S Dooph RT Kirk SL Mackie PM Hebert PDN (2005) Critical factors for assembling a high volume of DNA barcodes. Philosophical Transactions of the Royal Society of London – Series B, Biological Sciences 360: 1959–1967. 10.1098/rstb.2005.1727 16214753 PMC1609220

[B17] Hall TA (1999) BioEdit: a user-friendly biological sequence alignment editor and analysis program for Windows 95/98/NT. Nucleic Acids Symposium Series 41: 95–98.

[B18] Ivanova NV deWaard JR Hebert PDN (2006) An inexpensive, automation-friendly protocol for recovering high quality DNA. Molecular Ecology Resources 6: 998–1002. 10.1111/j.1471-8286.2006.01428.x

[B19] Kandul NP (1997) The karyology and the taxonomy of the blue butterflies of the genus *Agrodiaetus* Hübner [1822] from the Crimea (Lepidoptera: Lycaenidae). Atalanta 28: 111–119.

[B20] Kandul NP Lukhtanov VA Dantchenko AV Coleman JWS Sekercioglu CH Haig D Pierce NE (2004) Phylogeny of *Agrodiaetus* Hübner 1822 (Lepidoptera: Lycaenidae) Inferred from mtDNA Sequences of *COI* and *COII* and Nuclear Sequences of *EF1*-*α*: Karyotype Diversification and Species Radiation. Systematic Biology 53(2): 278–298. 10.1080/10635150490423692 15205053

[B21] Kandul NP Lukhtanov VA Pierce NE (2007) Karyotypic diversity and speciation in *Agrodiaetus* butterflies. Evolution 61(3): 546–559. 10.1111/j.1558-5646.2007.00046.x 17348919

[B22] Lukhtanov VA (1989) Karyotypes of some blue butterflies of the *Agrodiaetus* species groups. Annales Entomologici Fennici 55: 137–144.

[B23] Lukhtanov VA (2015) The blue butterfly Polyommatus (Plebicula) atlanticus (Lepidoptera, Lycaenidae) holds the record of the highest number of chromosomes in the non-polyploidy eukaryotic organisms. Comparative Cytogenetics 9(4): 683–690. 10.3897/CompCytogen.v9i4.5760 26753083 PMC4698580

[B24] Lukhtanov VA Dantchenko AV (2002a) Principles of highly ordered metaphase I bivalent arrangement in spermatocytes of *Agrodiaetus* (Lepidoptera). Chromosome Research 10(1): 5–20. 10.1023/A:1014249607796 11863071

[B25] Lukhtanov VA Dantchenko AV (2002b) Descriptions of new taxa of the genus *Agrodiaetus* Hübner, [1822] based on karyotype investigation (Lepidoptera, Lycaenidae). Atalanta 33(1/2): 81–107, 224–225.

[B26] Lukhtanov VA Dantchenko AV Kandul NP (1997) Die Karyotypen von Polyommatus (Agrodiaetus) damone damone und P. (A.) damocles rossicus nebst einigen Problemen bei *Agrodiaetus* (Lepidoptera: Lycaenidae). Nachrichten des Entomologischen Vereins Apollo Supplement 16: 43–48.

[B27] Lukhtanov VA Kandul NP Plotkin JB Dantchenko AV Haig D Pierce NE (2005) Reinforcement of pre-zygotic isolation and karyotype evolution in *Agrodiaetus* butterflies. Nature 436(7049): 385–389. 10.1038/nature03704 16034417

[B28] Lukhtanov VA Vila R Kandul NP (2006) Rearrangment of the *Agrodiaetus dolus* species group (Lepidoptera, Lycaenidae) using a new cytological approach and molecular data. Insect and Systematic and Evolution 37(3): 325–334. 10.1163/187631206788838563

[B29] Lukhtanov VA Shapoval NA Dantchenko AV (2008) *Agrodiaetus shahkuhensis* sp. n. (Lepidoptera, Lycaenidae), a cryptic species from Iran discovered by using molecular and chromosomal markers. Comparative Cytogenetics 2(2): 99–114.

[B30] Lukhtanov VA Sourakov A Zakharov EV Hebert PDN (2009) DNA barcoding Central Asian butterflies: increasing geographical dimension does not significantly reduce the success of species identification. Molecular Ecology Resources 9: 1302–1310. 10.1111/j.1755-0998.2009.02577.x 21564901

[B31] Lukhtanov VA Shapoval NA Dantchenko AV (2014) Taxonomic position of several enigmatic Polyommatus (Agrodiaetus) species (Lepidoptera, Lycaenidae) from Central and Eastern Iran: insights from molecular and chromosomal data. Comparative Cytogenetics 8(4): 313–322. 10.3897/CompCytogen.v8i4.8939 25610545 PMC4296718

[B32] Lukhtanov VA Shapoval NA Anokhin BA Saifitdinova AF Kuznetsova VG (2015) Homoploid hybrid speciation and genome evolution via chromosome sorting. Proceedings of the Royal Society B – Biological Sciences 282(1807): 20150157 10.1098/rspb.2015.0157 PMC4424644 25925097

[B33] Lukhtanov VA Sourakov A Zakharov EV (2016) DNA barcodes as a tool in biodiversity research: testing pre-existing taxonomic hypotheses in Delphic Apollo butterflies (Lepidoptera, Papilionidae). Systematics and Biodiversity 14(6): 599–613. 10.1080/14772000.2016.1203371

[B34] Maddison WP Maddison DR (2015) Mesquite: a modular system for evolutionary analysis. Version 3.04. http://mesquiteproject.org

[B35] Przybyłowicz Ł Lukhtanov V Lachowska-Cierlik D (2014) Towards the understanding of the origin of the Polish remote population of Polyommatus (Agrodiaetus) ripartii (Lepidoptera: Lycaenidae) based on karyology and molecular phylogeny. Journal of Zoological Systematics and Evolutionary Research 52(1): 44–51. 10.1111/jzs.12040

[B36] Rambaut A Drummond AJ Xie D Baele G Suchard MA (2018) Posterior summarisation in Bayesian phylogenetics using Tracer 1.7. Systematic Biology 67: 901–904. 10.1093/sysbio/syy032 29718447 PMC6101584

[B37] Ronquist F Teslenko M van der Mark P Ayres DL Darling A Hohna S Larget B Liu L Suchard MA Huelsenbeck JP (2012) MrBayes 3.2: efficient Bayesian phylogenetic inference and model choice across a large model space. Systematic Biology 61: 539–542. 10.1093/sysbio/sys029 22357727 PMC3329765

[B38] Staudinger O (1899) Ueber die Arten and Formen der *Lycaena damon*-Gruppe. Deutsche Entomologische Zeitschrift Iris 12: 137–155.

[B39] Vershinina AO Lukhtanov VA (2010) Geographical distribution of the cryptic species *Agrodiaetus alcestis alcestis*, *A. alcestis karacetinae* and *A. demavendi* (Lepidoptera, Lycaenidae) revealed by cytogenetic analysis. Comparative Cytogenetics 4(1): 1–11. 10.3897/compcytogen.v4i1.21

[B40] Vershinina AO Anokhin BA Lukhtanov VA (2015) Ribosomal DNA clusters and telomeric (TTAGG)n repeats in blue butterflies (Lepidoptera, Lycaenidae) with low and high chromosome numbers. Comparative Cytogenetics 9(2): 161–171. 10.3897/CompCytogen.v9i2.4715 26140159 PMC4488964

[B41] Vershinina AO Lukhtanov VA (2017) Evolutionary mechanisms of runaway chromosome number change in *Agrodiaetus* butterflies. Scientific Reports 7: e8199. 10.1038/s41598-017-08525-6 PMC5557896 28811556

[B42] Vishnevskaya MS Saifitdinova AF Lukhtanov VA (2016) Karyosystematics and molecular taxonomy of the anomalous blue butterflies (Lepidoptera, Lycaenidae) from the Balkan Peninsula. Comparative Cytogenetics 10(5): 1–85. 10.3897/CompCytogen.v10i5.10944 PMC5220643 28105291

[B43] Vodolazhsky D Stradomsky B (2012) Comparative genital and molecular genetic analysis of blue butterflies (Lepidoptera: Lycaenidae) of Rostov-on-Don area. In: Troitsky AV Rusin LY Aleoshin VV (Eds) Molecular Phylogenetics.Moscow, Torus Press, 70–71.

[B44] Vodolazhsky DI Yakovlev R Stradomsky B (2011) Study of taxonomic status of some specimens of subgenus Agrodiaetus (Lepidoptera: Lycaenidae: *Polyommatus*) from Western Mongolia based on mtDNA markers. Caucasian Entomological Bulletin 7(1): 81–82. 10.23885/1814-3326-2011-7-1-81-82

[B45] Wiemers M (2003) Chromosome Differentiation and the Radiation of the Butterfly subgenus Agrodiaetus (Lepidoptera: Lycaenidae: *Polyommatus*) a Molecular Phylogenetic Approach. PhD Thesis. University of Bonn, Bonn, 203 pp http://hss.ulb.uni-bonn.de/2003/0278/0278.htm

